# Case Report: Biliary hemorrhage by intrahepatic pseudoaneurysm and asymptomatic right coronary artery pseudoaneurysm in a patient with STAT3 hyper IgE syndrome

**DOI:** 10.3389/fimmu.2025.1601776

**Published:** 2025-05-26

**Authors:** Daiki Fujita, Masahiro Ueki, Hiroshi Yamanaka, Kota Watanabe, Satoshi Yakuwa, Atsushi Manabe, Masafumi Yamada

**Affiliations:** ^1^ Department of Pediatrics, Hokkaido University Hospital, Sapporo, Japan; ^2^ Department of Pediatrics, Obihiro-Kosei General Hospital, Obihiro, Japan; ^3^ Department of Food and Human Wellness, Rakuno Gakuen University, Ebetsu, Japan

**Keywords:** hyper IgE syndrome, *STAT3*, biliary hemorrhage, intrahepatic pseudoaneurysm, coronary pseudoaneurysm

## Abstract

STAT3-hyper IgE syndrome (STAT3-HIES) is a primary immunodeficiency disorder caused by dominant-negative mutations in *STAT3*, leading to defects in Th17 cell differentiation, immune regulation, and tissue repair. Patients are susceptible to recurrent infections and vascular abnormalities, such as vasculopathy and pseudoaneurysms. While involvement of cerebral, bronchial, and coronary arteries has been reported, hepatic artery involvement is rare. We describe a 25-year-old woman with genetically confirmed STAT3-HIES who presented with biliary hemorrhage secondary to a ruptured hepatic pseudoaneurysm. Emergency transcatheter arterial embolization successfully controlled the hemorrhage, and the patient was discharged without complications. Systemic vascular screening revealed an asymptomatic right coronary artery dilation, necessitating medical management with statin therapy. This case highlights hepatic pseudoaneurysm as a rare but life-threatening vascular complication in STAT3-HIES. Given the potential for multi-organ vasculopathy, systemic vascular screening by contrast-enhanced CT or MRI is crucial for early detection and management. Further research is needed to elucidate the mechanisms underlying vasculopathy in STAT3-HIES and establish optimal screening strategies to improve patient outcomes.

## Introduction

Inborn errors of immunity (IEIs) are diseases caused by pathogenic variants in genes that affect immune cell function (1). Primary immunodeficiency (PID) is one of the IEIs susceptible to infections, which can sometimes lead to severe or disseminated infections. STAT3-hyper IgE syndrome (STAT3-HIES) is a type of PID caused by dominant-negative pathogenic variants in *STAT3*. Patients with STAT3-HIES typically present with the classic triad of cold abscesses, elevated serum IgE levels, and refractory eczema beginning in the neonatal period (2). Impaired activation of STAT3 affects the JAK-STAT signaling pathway, such as Interleukin (IL)-6, IL-10, and IL-11 signaling. Consequently, this affects immunological activation including Th17 cell differentiation, vascular endothelial cell function, and tissue repair. Some patients with STAT3-HIES develop vasculopathy or pseudoaneurysms in the bronchial, coronary, or cerebral arteries due to endothelial fragility and impaired tissue repair (3, 4). However, reports of hepatic artery involvement are rare.

In this report, we describe a case of biliary hemorrhage due to the rupture of an intrahepatic pseudoaneurysm in a patient with STAT3-HIES and provide a review of the literature on vasculopathy and pseudoaneurysms in STAT3-HIES patients. Patients with STAT3-HIES have been reported to develop vasculopathy involving the aortic, intracranial, coronary, and pulmonary arteries. In addition, as demonstrated in our patient, vasculopathy may also affect abdominal arteries, including the hepatic artery.

## Case description

A 25-year-old woman with STAT3-HIES presented with a three-week history of worsening epigastric pain and vomiting. She was transported to the emergency hospital by ambulance and underwent endoscopy because of possible biliary tract obstruction by elevated levels of hepatobiliary enzymes, hyperbilirubinemia, and elevated pancreatic enzymes. Endoscopy showed biliary hemorrhage. Enhanced-contrast CT revealed an extravasation of hepatic artery suggesting ruptured hepatic pseudoaneurysm. She was subsequently transferred to our hospital for intravenous catheter treatment.

The patient had a history of intractable eczema since birth and recurrent viral and bacterial infections during infancy. At 7 months of age, she was hospitalized for pneumonia. At 1 year of age, she was clinically diagnosed with HIES based on recurrent infections, characteristic facial features, and elevated serum IgE levels, and she was started on sulfamethoxazole-trimethoprim prophylaxis. At age 9, genetic testing identified a *de novo* reported heterozygous dominant-negative variant, p.R382W, in *STAT3*, as previously described (5). At age 12, she experienced recurrent lung abscesses due to co-infection with MSSA and MRSA (**Supplementary Figures 1A, B**). Vancomycin treatment was not tolerated due to Redman syndrome, and a left lower lobe pneumonectomy was ultimately performed without complications. Pathology showed infection with both Staphylococcus and Aspergillus species. At 13, she developed MRSA pneumonia (**Supplementary Figures 1C, D**), which did not respond to arbekacin or panipenem-betamipron but improved with rifampicin based on susceptibility testing. With sulfamethoxazole-trimethoprim and fluconazole prophylaxis, she remained free of further infections. She had no history of smoking, alcohol use, significant trauma, or vasculitis-related diseases including Kawasaki disease.

**Figure 1:**
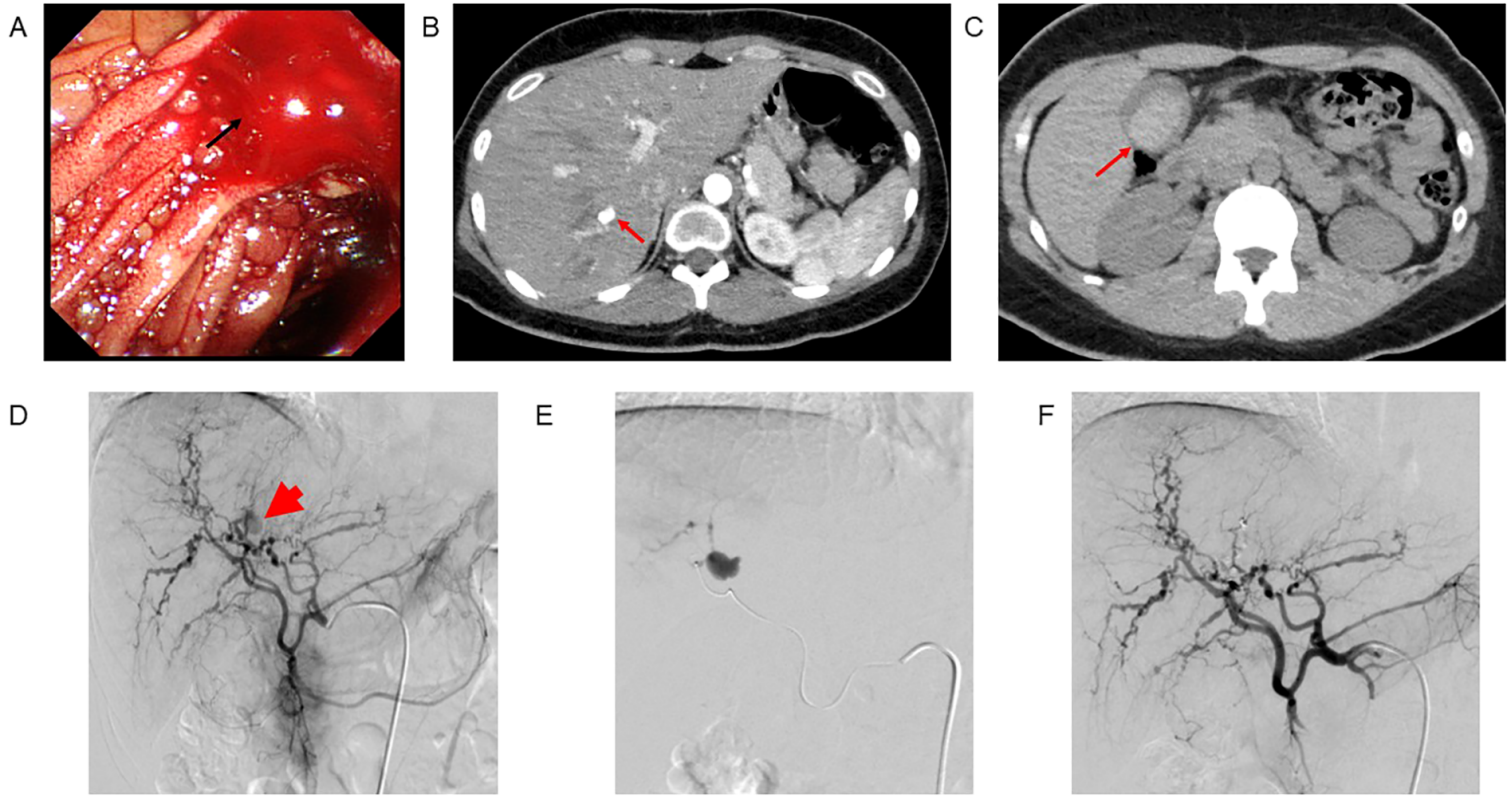
Images at the rupture of hepatic pseudoaneurysm and biliary hemorrhage in the patient with STAT3-HIES. Endoscopy revealed bleeding from the ampulla of Vater **(A)**. Contrast-enhanced CT showed an active hepatic bleeding **(B)** and highly resorbed area in the gallbladder likely representing a hematoma **(C)**. Angiography identified an intrahepatic pseudoaneurysm and active bleeding **(D)**. Catheter embolism was successfully performed, effectively controlling the bleeding **(E, F)**.

At 25 years old, she presented with epigastric pain and vomiting due to biliary hemorrhage. Laboratory findings showed normocytic anemia (Hb 8.7 g/dL), elevated hepatobiliary enzymes, hyperbilirubinemia, and elevated pancreatic enzymes, but no evidence of inflammation or coagulation abnormalities (**Supplementary Table 1**). Endoscopy showed bleeding from the ampulla of Vater (**Figure 1A**). Contrast-enhanced CT revealed a hematoma in the gallbladder, active bleeding from the hepatic artery (**Figures 1B, C**). The patient was diagnosed with biliary hemorrhage due to the rupture of a hepatic pseudoaneurysm and underwent emergency transcatheter arterial embolization (**Figures 1D-F**). She was discharged on the sixth day of hospitalization without further bleeding.

**Figure 2:**
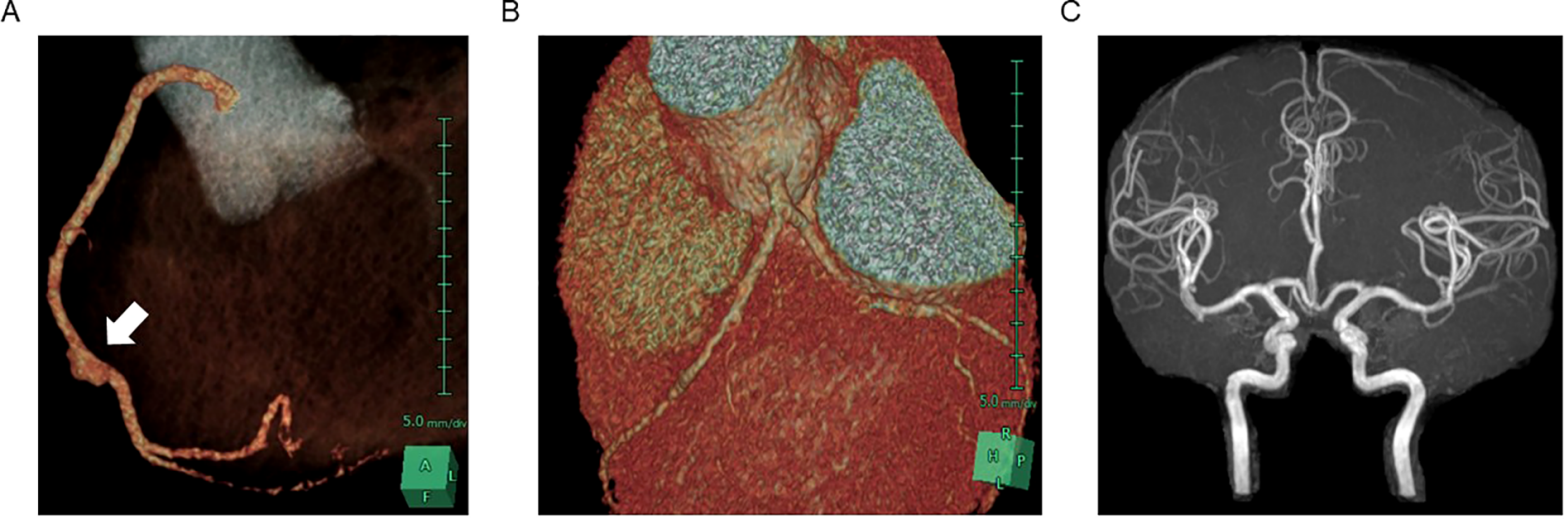
CT image of asymptomatic pseudoaneurysm in the right coronary artery. 3D reconstruction of contrast-enhanced CT image in coronary arteries demonstrated pseudoaneurysm in right coronary artery **(A)** and no dilation observed in left coronary arteries **(B)**. MR angiography showed no dilation in cerebral arteries **(C)**.

A few days later, coronary CT angiography and brain MRI with MR angiography (MRA) were performed for possible complication with asymptomatic vasculopathy. Coronary CT angiography showed spindle-shaped dilation in the right coronary artery, while no abnormalities were detected in the left coronary artery (**Figures 2A, B**). Brain MRI and MRA revealed no abnormalities (**Figure 2C**). She received rosuvastatin calcium therapy to prevent the atherosclerosis and has been recovering without any complications for 2 years.

We reviewed previous reports of vasculopathy in patients with STAT3-HIES including autosomal dominant or sporadic HIES without consanguinity (3, 4, 6–14) (**Table 1**). Reported vascular abnormalities include aneurysms, arterial dilation, cerebral infarction, and imaging findings indicative of tissue ischemia and vascular injury. A total of 33 patients (20 males, 13 females) across 11 reports were included. The most frequently affected vessels were the cerebral arteries (22 patients), followed by the coronary arteries (15 patients). Among patients with cerebrovascular abnormalities, 9 (40.9%) had concomitant coronary artery dilation. Five patients (14.3%) were diagnosed in childhood, with the youngest being 2 years old. The median age at diagnosis of vasculopathy was 27.9 years. The median age at diagnosis for cerebral arterial dilation was 28 years, and for coronary arterial dilation, 29 years.

**Table 1:** STAT3-hyper IgE syndrome patients with vasculopathy.

case	origin	sex	vasculopathy onset (age)	site			detail of vasculopathy	previous infection	surgical or other treatment	STAT3 variant	HIES score	ref.
				cerebral	coronary	other						
1	N.D	M	3	+			pseudoaneurysm	ear infection		V637M	N.D	(11)
2	N.D	F	10	+			infarction	N.D		N.S (sp)	36	(3)
3	Algeria	M	18	+			moderate WMH	pneumonia, dermatitis		R382W	74	(4)
4	France	M	19	+			mild WMH	pneumonia, dermatitis		K709E	77	(4)
5	France	M	19	+			moderate WMH	pneumonia, dermatitis		R382W	37	(4)
6	France	M	19	+			severe WMH	pneumonia, dermatitis		N472D	55	(4)
7	France	F	22	+	+		mild WMH, CA	pneumonia, dermatitis		R382W	86	(4)
8	France	M	23	+	+		mild WMH, CA	pneumonia, dermatitis		R382W	68	(4)
9	France	F	24	+	+		severe WMH, CA	pneumonia, dermatitis		R382W	73	(4)
10	France	M	25	+	+		moderate WMH, CD	pneumonia, dermatitis		R382W	84	(4)
11	France	F	26	+			mild WMH	pneumonia, dermatitis		V637M	73	(4)
12	France	F	28	+			severe WMH	pneumonia, dermatitis		R382W	73	(4)
13	France	M	29	+	+		moderate WMH, CA, LGE	pneumonia, dermatitis		R382Q	67	(4)
14	France	M	30	+	+		mild WMH, CA, LGE	pneumonia, dermatitis		Vdel463	64	(4)
15	France	M	30	+	+		moderate WMH, CD	dermatitis		I568F	41	(4)
16	France	M	37	+	+		moderate WMH, CD	pneumonia, dermatitis		V637M	74	(4)
17	France	M	38	+	+		moderate WMH, CA, CD, LGE	pneumonia, dermatitis		K642E	66	(4)
18	Pakistan	F	38	+			mild WMH	pneumonia, dermatitis		Vdel463	79	(4)
19	France	M	39	+			mild WMH	pneumonia, dermatitis		F384L	72	(4)
20	France	F	45	+			severe WMH	pneumonia, dermatitis		I665N	65	(4)
21	N.D	F	54	+			thromboembolism	N.D		N.S (AD)	N.D	(3)
22	N.D	F	50	+			aneurysm (rupture)	N.D		N.S (AD)	N.D	(13)
24	N.D	M	13		+		CD	N.D		N.S (AD)	65	(9)
25	Morocco	F	19		+		LGE	pneumonia, dermatitis		T714I	78	(4)
26	Caucasian	M	26		+		thromboembolism	pneumonia, dermatitis	HCT	R382W	76	(14)
27	N.D	F	30		+		CA	N.D		N.S (sp)	N.D	(3)
28	Caucasian	M	43		+		CA, CD	aspergillus pneumonia	pneumonectomy	N.S (sp)	79	(7)
29	N.D	M	48		+		CA	pneumonias, sinusitis, disseminated histoplasmosis, cryptococcal meningitis, mucocutaneous candidiasis	pneumonectomy	N.S (AD)	79	(7)
30	N.D	M	27			+	aortic aneurysm (rupture)	pneumonia, sinusitis,dpyelonephritis	nephrectomy	N.S (AD)	N.D	(10)
31	N. D	F	N.D			+	aortic aneurysm (rupture)	N.D		N.S (AD)	N.D	(3)
32	N. D	M	56			+	pulmonary pseudoaneurysm	pneumonia, sinusitis, Aspergillomas	pneumonectomy	N.S (AD)	N.D	(12)
33	N. D	F	2			+	bronchial artery	pneumonia		N.S (sp)	N.D	(6)
34	N. D	M	30			+	bronchial artery	N.D		N.S (sp)	N.D	(8)
35	Japanese	F	25		+	+	intrahepatic bleeding, CA	pneumonia, dermatitis	pneumonectomy	R382W	66	present

N.D, no data; AD, autosomal dominant; WMH, white matter hyperintensities (lacunar lesions suggestive of ischemic infarcts); CA, coronary aneurysm; CD, coronary dilation; LGE, late gadolinium enhancement; HCT, hematopoietic cell transplantation; N.S, not specified; sp sporadic.

## Discussion

In this report, we present a case of biliary hemorrhage due to hepatic pseudoaneurysm rupture in a STAT3-HIES patient, along with an asymptomatic pseudoaneurysm in the right coronary artery.

In STAT3-HIES, immunodeficiency is primarily due to defective IL-6 signaling, which plays a crucial role in Th17 cell differentiation. Th17 cell deficiency leads to reduced IL-17 production, impairing neutrophil migration and activation. Consequently, neutrophils fail to migrate effectively to infection sites, leading to inadequate immune responses against *Staphylococcus aureus* and fungal infections (2). Managing drug resistant *Staphylococcus aureus* infections, such as MRSA, is critical in patients with STAT3-HIES. Although vancomycin is the first-line therapy, its use can be intolerable by Redman syndrome, as observed in this case. Combination therapy with rifampicin and vancomycin or clindamycin has been reported effective for MRSA pneumonia (15). However, as demonstrated in this case, rifampicin monotherapy may also be effective, although careful monitoring for the emergence of drug-resistant strains is necessary.

Impaired tissue repair and angiogenesis, involving FGF1, FGF2, TGF-β, VEGF, and HIF-1α, has been implicated in vasculopathy and pseudoaneurysm formation in STAT3-HIES (16). Additionally, eosinophilic inflammation and Th17 deficiency may contribute to vascular pathology (3, 17). As observed in eosinophilic granulomatosis with polyangiitis, persistent eosinophilia may be associated with vasculitis. Furthermore, in a mouse model, IL-17 neutralization led to increased aneurysm severity and fatal rupture, although the underlying mechanism remains unclear. T cell–mediated inflammation may contribute to severe aneurysm formation, as increased T cell infiltration in the aneurysm was observed in these mice (4). Previous report showed the thickness of coronary artery wall and increased vessel area in patients with STAT3-HIES (18). However, the risk factors of vasculopathy remain unclear. Although vasculopathy is relatively common in adult patients, the factors such as hypertension, atherosclerosis, and infections remain uncertain (3, 17). Furthermore, our review of previous cases identified 5 pediatric patients, including 2 adolescents, suggesting that aging, hypertension, atherosclerosis, and recurrent infections are not the sole risk factor (**Table 1**). Identification of risk factors, especially for children, is essential, to improve the prognosis, although we could not determine them through our review. The most commonly affected arteries are the cerebral and coronary arteries, with severe complications such as aortic aneurysm rupture, hemoptysis, and myocardial infarction reported. Notably, vasculopathy could appear more frequent in males, which has not been previously highlighted (4, 17).

STAT3-HIES vasculopathy can involve any large or medium-sized artery, since the hepatic artery was also affected in this case. Therefore, systemic vascular screening is crucial. Although there are no standardized vascular screening guidelines for STAT3-HIES, early detection and intervention (e.g., medical therapy, catheter-based procedures, or surgery) are essential. In our review, screening for vascular involvement was performed using MRI or contrast-enhanced CT. A total of ten patients, including the present case, exhibited vasculopathy affecting two or more arteries. These findings suggest that patients who develop vasculopathy should undergo comprehensive whole-body vascular screening. Regarding treatment, anticoagulants and lipid-lowering agents, such as aspirin or statins, may be considered, although standardized treatment guidelines have not yet been established. In this case, rosuvastatin calcium was administered to prevent atherosclerosis and to reduce the risk of cardiovascular and cerebrovascular complications (19). Further patient-based research is needed to elucidate pathophysiological mechanisms, risk factors, optimal screening strategies and treatments to improve patient outcomes.

## Data Availability

The raw data supporting the conclusions of this article will be made available by the authors, without undue reservation.

## Supplementary material

Supplementary Figure 1 Images of the recurrent pulmonary infections. At age 9, the patient developed lung abscess, which was ultimately treated with surgical resection of left lung lobe, in the left lung by chest X ray **(A)** and CT **(B)** images. At age 12, the patient developed right pneumonia by Methicillin-resistant *Staphylococcus aureus*, successfully treated with rifampicin. Infiltration in the right lung was confirmed by chest X ray **(C)** and CT **(D)**.
