# Supplementary material for: Case Report: Biliary hemorrhage by intrahepatic pseudoaneurysm and asymptomatic right coronary artery pseudoaneurysm in a patient with STAT3 hyper IgE syndrome
Source: Front Immunol. 2025 May 26;16:1601776. doi: 10.3389/fimmu.2025.1601776 (PMC12146391; doi:10.3389/fimmu.2025.1601776)
Supplement: Supplementary file 1 [file Table1.docx]

Supplement Table 1 Laboratory findings before and at onset of the rupture of pseudoaneurism in hepatic artery

|  | Before onset | onset |
| --- | --- | --- |
| **White blood cell count (x10^3^ cells/μL)** | 3.7 | 4.2 |
| **Neutrophils (%)** | 42 | 36 |
| **Lymphocytes (%)** | 39 | 42 |
| **CD3+ (% in lymphocytes)** | 81.1 | No data |
| **CD4+ (% in lymphocytes)** | 42.3 | No data |
| **CD4+CD45RA+ (% in lymphocytes)** | 34.3 | No data |
| **CD8+ (% in lymphocytes)** | 46.0 | No data |
| **CD20** | 7.0 | No data |
| **Eosinophils (%)** | 13 | 14 |
| **Red blood cells count (x10^6^ cells/μL)** | 4.13 | 3.02 |
| **Hemoglobin (g/L)** | 119 | 87 |
| **Hematocrit (%)** | 37.1 | 27.6 |
| **Platelet count (x10^3^ /μL)** | 192 | 195 |
| **Prothrombin time (% of normal)** | 113 | 109 |
| **APTT (sec)** | 32.5 | 30.7 |
| **Fibrinogen (g/L)** | 2.30 | No data |
| **D-dimer (μg/mL)** | 0.8 | No data |
| **FDP (μg/mL)** | No data | 5.3 |
| **AST (U/L)** | 26 | 400 |
| **ALT (U/L)** | 21 | 660 |
| **ALP (U/L)** | 40 | 205 |
| **LDH (U/L)** | 219 | 273 |
| **GTP (U/L)** | 11 | 217 |
| **T-Bil (mg/L)** | 6 | 14 |
| **D-Bil (mg/L)** | No data | 7 |
| **Amy (U/L)** | No data | 145 |
| **Lipa (IU/L)** | No data | 127 |
| **BUN (mg/L)** | 145 | 71 |
| **Cre (mg/L)** | 9.2 | 6.2 |
| **CRP** | <0.1 | 0.3 |
| **IgG (g/L)** | 23.31 | No data |
| **IgA (g/L)** | 2.48 | No data |
| **IgM (g/L)** | 0.69 | No data |
| **IgE (U/mL)** | 41996 | No data |
