# Supplementary figures and images for: Case Report: Biliary hemorrhage by intrahepatic pseudoaneurysm and asymptomatic right coronary artery pseudoaneurysm in a patient with STAT3 hyper IgE syndrome
Source: Front Immunol. 2025 May 26;16:1601776. doi: 10.3389/fimmu.2025.1601776 (PMC12146391; doi:10.3389/fimmu.2025.1601776)

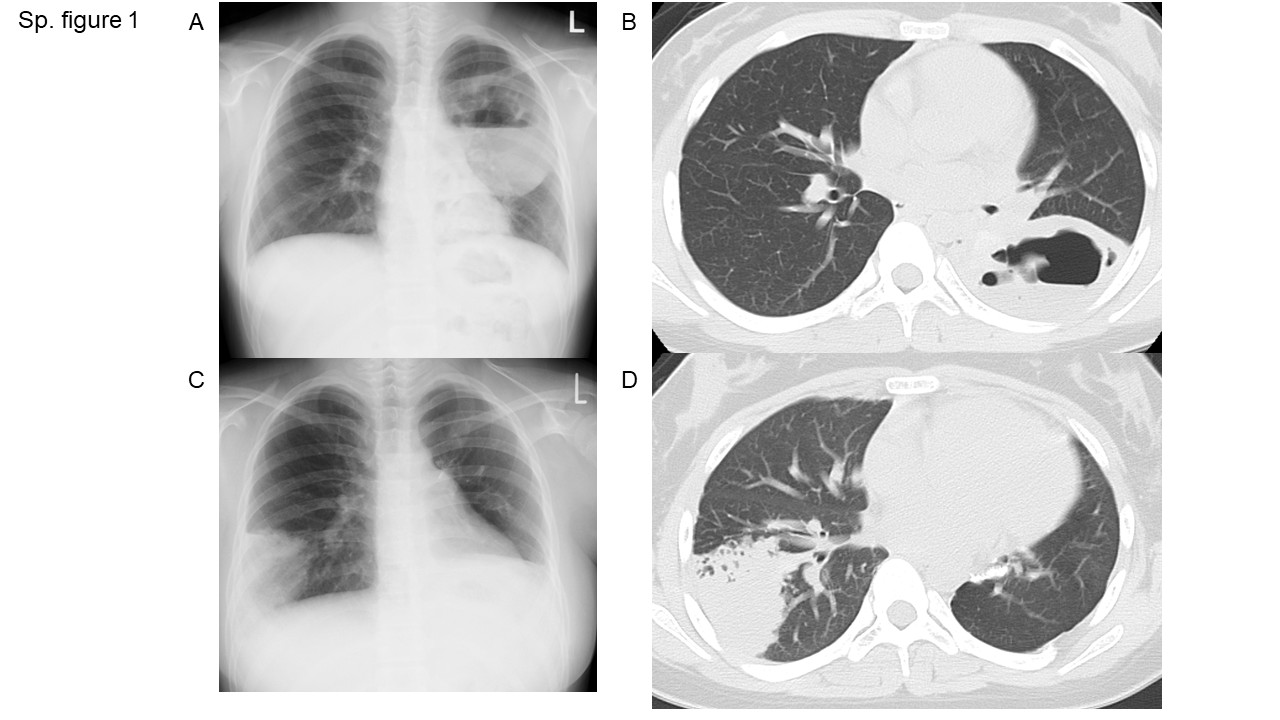

Supplement: Supplementary Figure 1 — Images of the recurrent pulmonary infections. At age 9, the patient developed lung abscess, which was ultimately treated with surgical resection of left lung lobe, in the left lung by chest X ray (A) and CT (B) images. At age 12, the patient developed right pneumonia by Methicillin-resistant Staphylococcus aureus, successfully treated with rifampicin. Infiltration in the right lung was confirmed by chest X ray (C) and CT (D). [file Image1.jpeg]
